# Supplementary material for: The causal role of circulating inflammatory markers in osteoporosis: a bidirectional Mendelian randomized study
Source: Front Immunol. 2024 Jul 18;15:1412298. doi: 10.3389/fimmu.2024.1412298 (PMC11291241; doi:10.3389/fimmu.2024.1412298)
Supplement: Supplementary file 1 [file DataSheet_1.zip › supplementary material/OSTEOPOROSIS-CIM/GCST90274816.tsv.gz_forest.pdf]

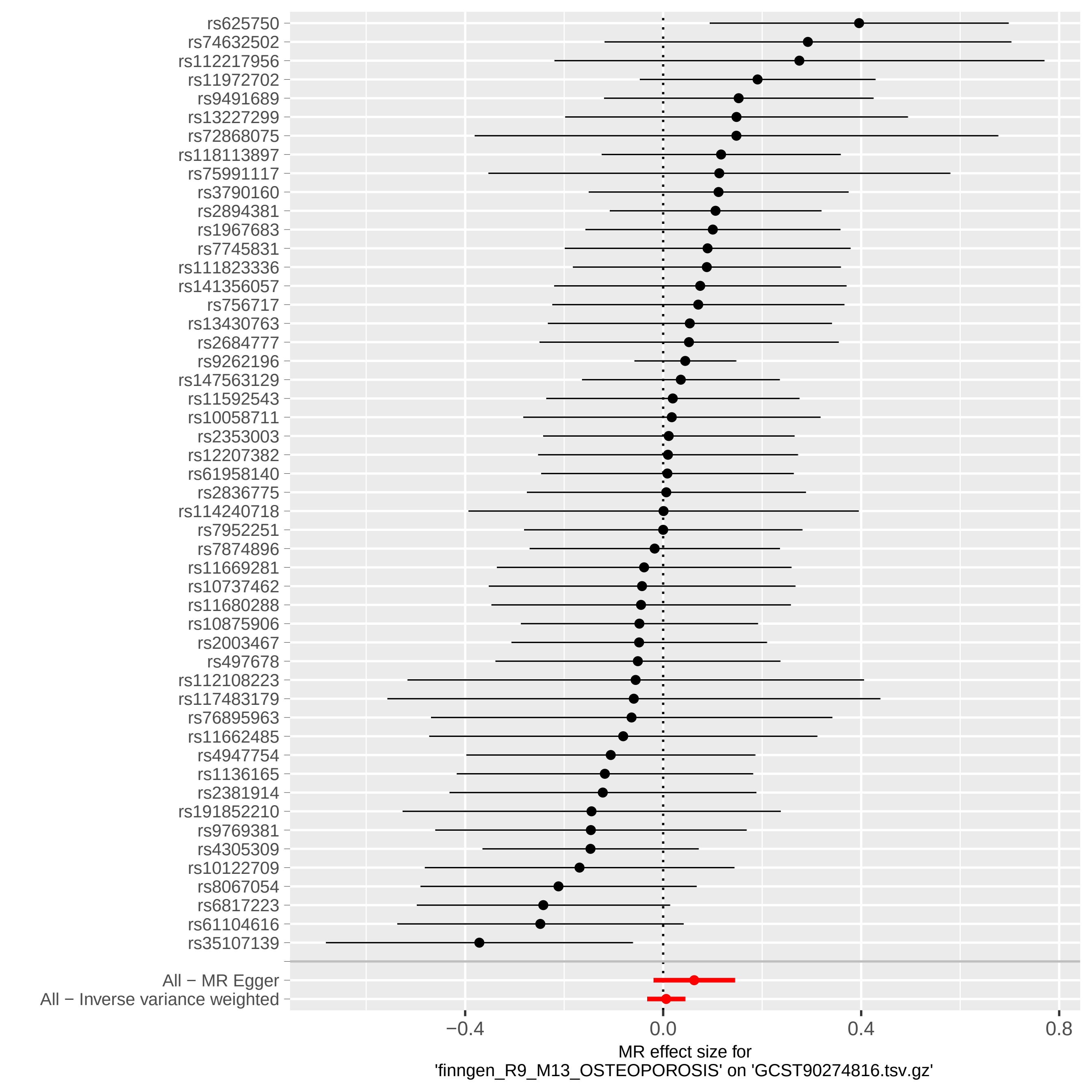
rs625750
rs74632502
rs112217956
rs11972702
rs9491689
rs13227299
rs72868075
rs118113897
rs75991117
rs3790160
rs2894381
rs1967683
rs7745831
rs111823336
rs141356057
rs756717
rs13430763
rs2684777
rs9262196
rs147563129
rs11592543
rs10058711
rs2353003
rs12207382
rs61958140
rs2836775
rs114240718
rs7952251
rs7874896
rs11669281
rs10737462
rs11680288
rs10875906
rs2003467
rs497678
rs112108223
rs117483179
rs76895963
rs11662485
rs4947754
rs1136165
rs2381914
rs191852210
rs9769381
rs4305309
rs10122709
rs8067054
rs6817223
rs61104616
rs35107139
All − MR Egger
All − Inverse variance weighted
−0.4
0.0
0.4
0.8
MR effect size for
'finngen_R9_M13_OSTEOPOROSIS' on 'GCST90274816.tsv.gz'
